# Evaluation of Electrolyte Imbalance in Patients With Traumatic Brain Injury Admitted in the Central ICU of a Tertiary Care Centre: A Prospective Observational Study

**DOI:** 10.7759/cureus.17517

**Published:** 2021-08-28

**Authors:** Sandeep Dey, Ramesh Kumar, Abhijit Tarat

**Affiliations:** 1 Department of Anaesthesiology, Jorhat Medical College and Hospital, Jorhat, IND; 2 Department of Anaesthesiology, Assam Medical College and Hospital, Dibrugarh, IND

**Keywords:** traumatic brain injury, hyponatraemia, hypokalaemia, hypomagnesemia, hypoalbuminemia

## Abstract

Introduction

Electrolyte imbalance is a salient finding in traumatic brain injury which can derail their clinical course of recovery in physical and cognitive health while prolonging the hospital stay.

Objective

This study aims to understand the variation in electrolyte profile that occurs in traumatic brain injury patients which can help in better patient management.

Materials and method

50 trauma patients with head injury (Group A) and 50 patients without head injury (Group B) admitted in Central ICU (CICU) under the Department of Anaesthesiology, Assam Medical College and Hospital (AMCH) were selected and analysed with regard to their electrolyte variability.

Result

All trauma patients with head injury developed an imbalance to one or more electrolytes. Then mean electrolyte level in trauma patients with a head injury and in trauma patients without head injury were 139.3±7.45 vs 143.65±8.89, p<0.05 (sodium), 3.49±0.44 vs 3.88±0.49, p<0.05 (potassium), 7.81±0.5 vs 8.9± 0.35, p<0.05 (calcium) and 2±0.33 vs 2.47±0.41, p<0.05 (magnesium) respectively. Also, patients in the head injury group had a higher incidence of hypoalbuminemia than patients without head injury 2.47±0.67 vs 2.83±0.74 (p<0.05).

Conclusion

We conclude that traumatic brain injury patients have a greater risk of electrolyte imbalance, viz. hyponatremia, hypokalaemia, hypocalcaemia as well as hypomagnesemia, and hypophosphatemia along with hypoalbuminemia.

## Introduction

Traumatic brain injury (TBI) - a silent epidemic, has emerged as a leading cause of mortality, morbidity, and fiscal loss in developing counties like India [1]. Road traffic accidents (RTA), the leading cause of TBI (around 60%), not only hits the most intellectual and economic class of 15-29 years but also causes loss to the Gross Domestic Product of the country to the tune of 1-3% [2]. In this study we aim to study the variation in various electrolyte profiles of TBI patients which can:

Prolong restoration of physical and cognitive health, increase the duration of hospital stay, upscale financial loss to the individual, state, and nation, and add up to the morbidity and, in severe cases, the mortality of the patient.

Lastly, the knowledge so gained can be useful to the medical fraternity for better fluid and electrolyte resuscitation in TBI patients as well as for further research.

Physiologically, body water and electrolytes are under a dynamic balance of central (neurohypophysis) and peripheral (renal, heart) neural and or hormonal control and any insult that jeopardizes this balance, viz. TBI can result in electrolyte imbalance.

Hyponatraemia (defined as serum sodium <135 mmol/L) is the most common finding after TBI, manifesting especially in those who are critically ill and maximally around two to seven days following trauma [3]. Causes may be cerebral salt wasting syndrome, syndrome of inappropriate antidiuretic hormone secretion (SIADH), or iatrogenic due to use of drugs (thiazide, loop diuretic like furosemide, osmotic agent like mannitol). Also as the duration of ICU stay lengthens pertinent to the severity of the injury or any cause, protein catabolism worsens; ensuing hypoalbuminemia, which can deteriorate the already set electrolyte imbalance (like hyponatremia) further [4]. Calcium ion which circulates in plasma bound to albumin may mimic a false low level in presence of hypoalbuminemia despite normal ionized calcium levels. The stress of TBI may provoke catecholamine release that can lead to hypokalaemia and leucocytosis. Similarly, the use of drugs like thiazide can land in hypomagnesemia and hypophosphatemia [5]. Both hyponatremia and hypocalcaemia have the potential to alter central nervous system (CNS) function from depression to confusion, and in acute state precipitating seizure. Severe hypocalcaemia can also manifest as laryngospasm or bronchospasm. Hypophosphatemia can result in impairment of diaphragmatic contractility resulting in difficulty in weaning from ventilation. Another electrolyte imbalance very common in ICU setup is hypomagnesemia which apart from being an independent risk factor for coronary heart disease and fatal ventricular arrhythmia is also frequently associated with hypocalcaemia and hypokalaemia [6-8].

With the above understanding, it is wise to choose appropriate fluid in TBI patients to prevent or treat any electrolyte imbalance at its earliest, while keeping a wise watch over nitrogen balance to halt its fall down the ladder of protein catabolism as much as feasible. 

## Materials and methods

The study was conducted in the Central ICU (CICU) of the Department of Anaesthesiology and Critical Care, Assam Medical College and Hospital (AMCH), Dibrugarh, India.

100 patients admitted to our CICU from 01.07.2018 to 30.06.2019 were enrolled in the study. They were divided into two groups of 50 each:

Group A: trauma with head injury (case)

Group B: trauma without head injury (control)

Patients in shock with a low Glasgow Coma Scale (GCS) score or any polytrauma patient where initial aggressive resuscitation was done outside CICU were excluded from the study. Patients who were referred from other hospitals or ICU were excluded. Patients at extremes of age (<12 years and >55 years) were excluded. Also, patients with chronic kidney disease, diabetic patients on insulin or oral hypoglycaemic agents (OHA), and patients of heart failure on diuretics were excluded from the study.

Blood investigations viz. complete blood count (CBC), random blood sugar (RBS), liver function test (LFT), kidney function test (KFT), serum sodium, potassium, calcium, magnesium, and phosphorus along with other investigations as necessary for the individual cases were sent routinely as par CICU protocol. Daily and precise monitoring of input-output was done including all losses via urine, stool, insensible water loss, drain/s, and suctioning. Patients were followed till discharge or demise from CICU and or till baseline values returned to normal. Results obtained were charted and analysed.

Daily measurement of serum sodium, potassium, calcium, magnesium, phosphate, chloride, and alternate day measurement of blood urea, serum creatinine, and albumin was done in both the groups (A and B). Both groups were compared with regard to their mean age, sex distribution, mean GCS at the time of initial evaluation, mean duration of their ICU stay, and variation in the mean electrolyte profile (of sodium, potassium, calcium, magnesium, phosphate, and chloride) during the course of their stay in the CICU. Also, both the groups were compared with regard to the variability in their mean serum albumin, urea and creatinine levels. Additionally, the variability in serum electrolytes (of sodium, potassium, calcium, and magnesium) in Group A has been depicted graphically and analysed. 

Statistical analysis

Statistical analysis was done using Microsoft Excel and Microsoft Word (Microsoft Corporation, Redmond, USA). Descriptive data were presented as mean ± standard deviation (SD). Categorical data were shown as percentage (%). Student's unpaired t-test was used to compare the difference between the mean of the two groups for the different variables under study. For all tests, a p-value of less than 0.05 was considered significant. 

## Results

Table 1 shows the age distribution, sex distribution, and the mean GCS of patients in groups A and B. In our study patients in Group A (mean age 42 years) were found to be significantly younger than patients in Group B (mean age 45 years). This is consistent with the finding that traumatic brain injury is more common in the younger age group. In our study, most of the patients were male in both groups. Also, we found in our study that patients with trauma with head injury (Group A) had low GCS than patients with trauma without head injury (Group B) and the difference between the two groups was statistically significant.

**Table 1:** Patient characteristics

CHARACTERISTIC	GROUP A	GROUP B	P-VALUE
MEAN AGE (IN YEARS)	42.46	45.72	0.0438
SEX DISTRIBUTION	MALE: 42, FEMALE: 8	MALE: 38, FEMALE: 12	-
MEAN GCS SCORE (MEAN ± SD)	5.46±1.431568	11.28±1.969564	<0.0001

Table 2 shows the causes of TBI in Group A and their CT Scan head findings. The most common mechanism of TBI in our study was road traffic accidents (RTA), followed by fall from height and physical assault. The most common CT scan head finding following both RTA and fall from height was subdural haemorrhage (SDH).

**Table 2:** Mechanism of injury RTA: road traffic accident SAH: subarachnoid haemorrhage SDH: subdural haemorrhage EDH: extradural haemorrhage DAI: diffuse axonal injury IPH: intraparenchymal haemorrhage IVH: intraventricular haemorrhage TBI: traumatic brain injury

MECHANISM OF TBI	CT SCAN HEAD FINDING	NUMBER OF PATIENTS	PERCENTAGE (%)
RTA	SAH	19	38
	SDH	23	46
	EDH	11	22
	DAI	5	10
	IPH	19	38
	IVH	15	30
FALL FROM HEIGHT	SAH	0	0
	SDH	6	12
	EDH	3	6
	DAI	0	0
	IPH, IVH	0	0
PHYSICAL ASSAULT	SAH	0	0
	SDH	0	0
	EDH	0	0
	DAI	2	4
	IPH, IVH	0	0

Table 3 gives a brief overview of the patients included in Group B ( trauma without head injury). In our study chest trauma was the most common finding in Group B, followed by blunt abdominal trauma and other trauma like femur fracture with fat embolism syndrome, pelvic fracture, etc. The absence of head injury in this group was confirmed by clinical evaluation and the absence of any evidence on radiological evaluation.

**Table 3:** Causes of trauma in Group B

DIAGNOSIS	NUMBER OF PATIENTS
CHEST TRAUMA	17
BLUNT ABDOMINAL TRAUMA	14
CRUSH INJURY LOWER LIMB	5
COMPARTMENT SYNDROME	3
SPINAL INJURY	2
OTHERS (fracture femur with fat embolism syndrome, pelvic fracture, etc.)	9

Table 4 shows the variability of the various variables under study in patients with traumatic brain injury (Group A), as their numerical value and as their relative percentage. In our study, the most common electrolyte imbalance in TBI patients was hyponatremia followed by hypokalaemia and hypocalcaemia. Magnesium deficiency was seen in almost 2/3rd of patients with traumatic brain injury. Almost all patients with traumatic brain injury developed hypoalbuminemia during their course of stay in the CICU.

**Table 4:** Findings on electrolyte, albumin, urea and creatinine study

ELECTROLYTE	EVENTS	NUMBER OF FINDINGS	PERCENTAGE
SODIUM	HYPO (<136 mmol/L)	41	82
	NORMAL (136 – 145 mmol/L)	49	
	HYPER (>145 mmol/L)	07	14
POTASSIUM	HYPO (<3.5 mmol/L)	39	78
	NORMAL (3.5 - 5 mmol/L)	48	
	HYPER (>5 mmol/L)	08	16
CALCIUM	HYPO (<8.5 mg/dL)	36	72
	NORMAL (8.5 – 10.5 mg/dL)	50	
	HYPER (>10.5 mg/dL)	0	0
MAGNESIUM	HYPO (<1.8 mg/dL)	33	66
	NORMAL (1.8 – 3 mg/dL)	43	
	HYPER (>3 mg/dL)	07	14
PHOSPHORUS	HYPO (<1 mmol/L)	22	44
	NORMAL (1 - 1.4 mmol/L)	44	
	HYPER (>1.4 mmol/L)	06	12
CHLORIDE	HYPO (< 98 mmol/L)	24	48
	NORMAL (98 – 106 mmol/L)	41	
	HYPER (> 106 mmol/L)	12	24
ALBUMIN	HYPO (<3.5 gm/dL)	50	100
	NORMAL (3.5 – 5.5 gm/dL)	43	
	HYPER (>5.5 gm/dL)	0	0
UREA	HYPO (< 3.6 mmol/L)	12	24
	NORMAL (3.6 - 7.1 mmol/L)	47	
	HYPER ( > 7.1 mmol/L)	10	20
CREATININE	HYPO (< 0.3 mg/dL)	18	36
	NORMAL (0.3 - 1 mg/dL)	48	
	HYPER (>1 mg/dL)	06	12

Table 5 gives a statistical analysis of the various variables under study between both groups. In our study, we found that patients with trauma with head injury (Group A) had statistically significant lower values for serum sodium, potassium, calcium, magnesium, phosphate, and also albumin than patients with trauma without head injury (Group B).

**Table 5:** Statistical analysis * Significant p-value (p<0.05)

PARAMETERS (NORMAL RANGE)	GROUP	MEAN±SD	P-VALUE	
SODIUM (136 - 145 mmol/L)	GROUP A	139.3 ± 7.45	0.01*	
	GROUP B	143.65 ± 8.89		
POTASSIUM (3.5 - 5 mmol/L)	GROUP A	3.49 ± 0.44	0.02*	
	GROUP B	3.88 ± 0.49		
CALCIUM (8.5 - 10.5 mg/dL)	GROUP A	7.81 ± 0.5	0.04*	
	GROUP B	8.9 ± 0.35		
MAGNESIUM (1.8 - 3 mg/dL)	GROUP A	2 ± 0.33	0.02*	
	GROUP B	2.47 ± 0.41		
PHOSPHATE (1 - 1.4 mmol/L)	GROUP A	3.24 ± 0.4	0.02*	
	GROUP B	3.41 ± 0.6		
CHLORIDE (98 - 106 mmol/L)	GROUP A	97.61 ± 5.16	0.02*	
	GROUP B	101.46 ± 1.68		
ALBUMIN (3.5 - 5.5 gm/dL)	GROUP A	2.47 ± 0.67	0.02*	
	GROUP B	2.83 ± 0.78		
UREA (3.6 - 7.1 mmol/L)	GROUP A	38.58 ± 25.41	0.13	
	GROUP B	35.94 ± 26.56		
CREATININE (0.3 - 1 mg/dL)	GROUP A	0.95 ± 0.84	0.8	
	GROUP B	1.07 ± 0.74		

The values of serum sodium, potassium, calcium and magnesium of Group A were further studied by line diagram, with the X-axis depicting the duration of CICU stay and the Y-axis depicting the absolute value of these variables. In all cases it was seen that as the duration of stay increased, there was a tendency towards electrolyte imbalance, viz. hyponatremia (serum sodium <135 mEq/L), hypokalaemia (serum potassium <3.5 mEq/L), hypocalcaemia (serum calcium <8 mmol/L), and hypomagnesemia (serum magnesium <1.7 mg/dL).

**Figure 1:**
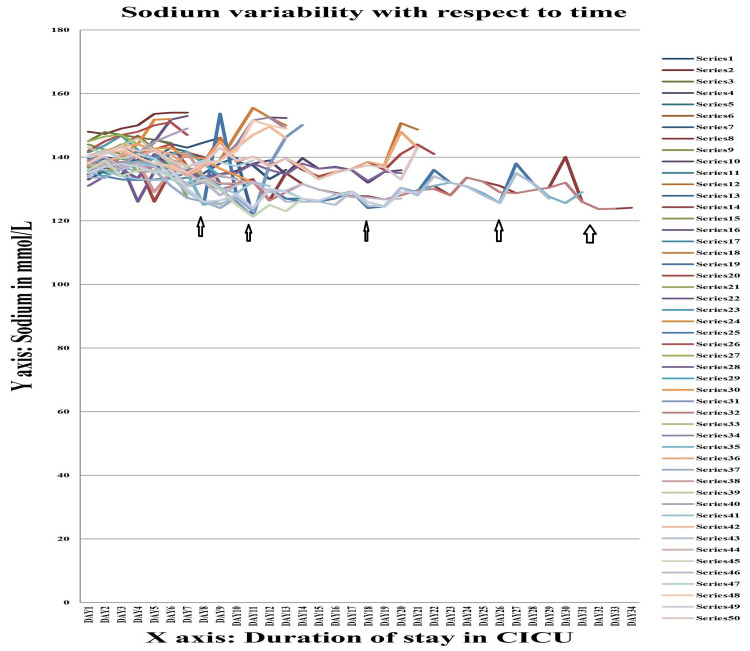
Serum sodium variability graph in Group A Each series denote the absolute value of serum sodium (in mmol/L) for a single individual during their stay in the Central ICU.

Figure 1 shows the variation in serum sodium in patients with trauma with head injury (Group A). During the initial days following trauma most patients had normal levels of serum sodium (136 - 145 mmol/L). By the seventh day of ICU stay some patients started developing hyponatraemia (serum sodium <136 mmol/L). This became more evident from the ninth to the 19th day. Those patients with ICU stay for three to four weeks and beyond were found to have the serum sodium level fluctuating towards lower level of normalcy, even after treatment. 

**Figure 2:**
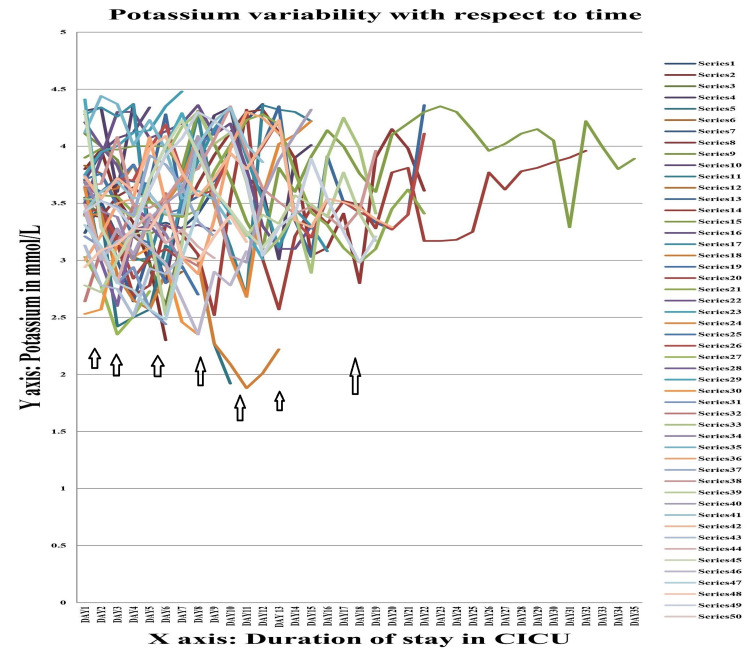
Serum potassium variability graph in Group A Each series denote the absolute value of serum potassium (in mmol/L) for a single individual during their stay in the Central ICU.

Figure 2 shows the variation in serum potassium in patients with trauma with head injury (Group A). In contrast to serum sodium, fall in serum potassium was evident as early as day 3. Hypokalaemia was found to persist as long as three weeks. After three weeks there was a tendency of a gradual recovery of serum potassium towards normalcy, with treatment.

**Figure 3:**
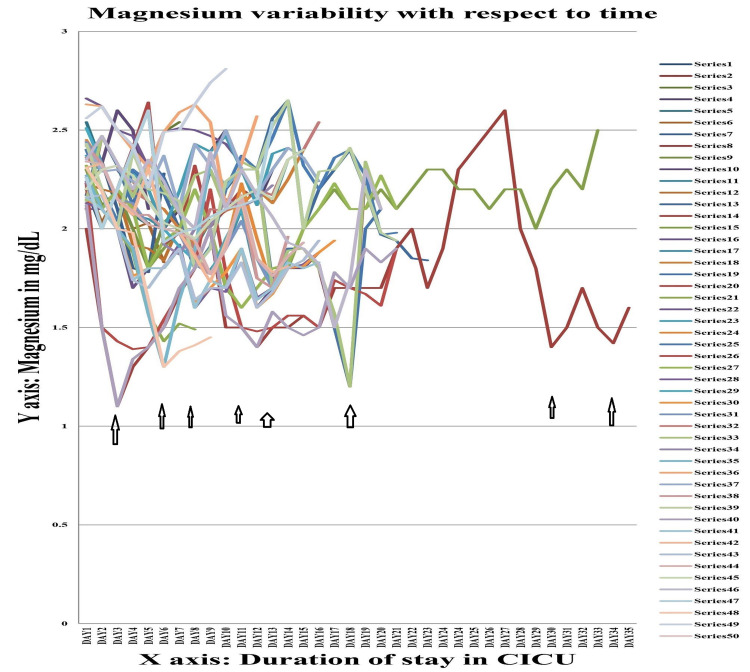
Serum magnesium variability graph in Group A Each series denote the absolute value of serum magnesium (in mg/dL) for a single individual during their stay in the Central ICU.

Figure 3 shows the variation in serum magnesium in patients with trauma with head injury (Group A). Fall in serum magnesium was evident as early as day 3. Hypomagnesemia was evident in most patients till second week, while in some it continued till third week. After third week, results were equivocal, while some patients responded to treatment, others continued to have hypomagnesaemia, even with treatment. 

**Figure 4:**
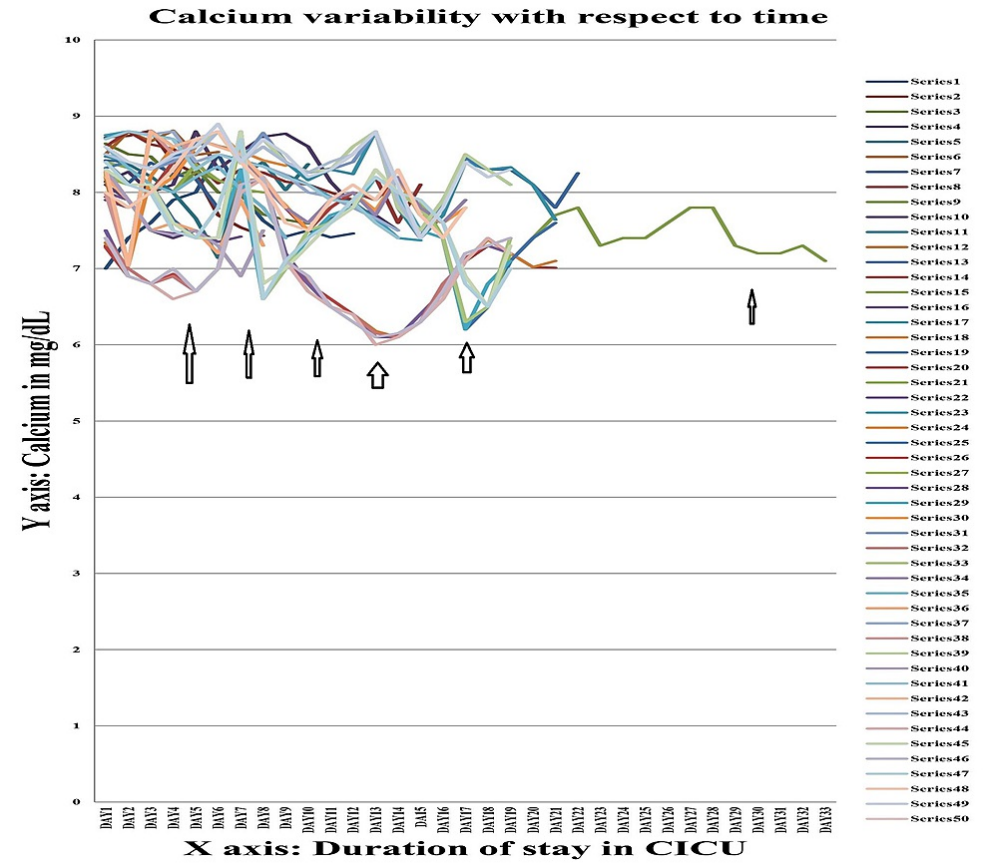
Serum calcium variability graph in group A Each series denote the absolute value of serum calcium (in mg/dL) for a single individual during their stay in the Central ICU.

Figure 4 shows the variation in calcium (in mg/dL) in patients with trauma with head injury (Group A). As with magnesium, fall in serum calcium was also evident as early as day 3 to day 5. In most patients, hypocalcaemia was mostly evident till second week, while in some cases it continued till the third week. Those patients whose ICU stay persisted beyond the third week, there was a tendency towards hypocalcaemia. 

Table 6 shows the mean duration of ICU stay between the two groups. In our study patients in Group A (trauma with head injury) had a statistically significant longer duration of ICU stay than patients in Group B (trauma without head injury). The longer duration of ICU stay explains in part the significant electrolyte imbalance seen in patients with traumatic brain injury.

**Table 6:** Average ICU stay

	GROUP A (MEAN ± SD)	GROUP B (MEAN ± SD)	P-VALUE
DAYS	13.38 ± 8.39	10.16 ± 7.18	0.04

Lastly, in Group A, blood urea was decreased in almost 1/4th of the patients, serum creatinine in almost 1/3rd while albumin in 100% of patients studied (Table 7). Nitrogen balance and electrolyte imbalance might have some correlation but that is way beyond the scope of this study and its findings.

**Table 7:** Nitrogen balance and electrolyte imbalance: Is there any correlation?

VARIABLE UNDER STUDY	RESULT	NUMBER OF PATIENTS	PERCENTAGE (%)
UREA	Decreased	12	24
CREATININE	Decreased	18	36
ALBUMIN	Decreased	50	100

## Discussion

In our study, we found that patients with traumatic brain injury are more likely to develop electrolyte imbalance than patients without head injury. This was consistent with the findings by Jain et al [9]. Most of the patients were male in both groups. The most common electrolyte imbalance in patients with traumatic brain injury was hyponatremia followed by hypokalaemia. This was consistent with the finding by Jain et al [9], Gupta et al [10], Adiga et al [11]. Other important electrolyte deficiencies noted were hypocalcaemia, hypophosphatemia, and hypomagnesemia. This was consistent with the study by Gupta et al [10], Suman S et al [12].

Calcium, magnesium, and phosphorus are amongst the least routinely measured electrolytes but bear high weightage as far as patient outcome is concerned. Almost every patient developed hypoalbuminemia during their course of stay in ICU. This is explained by three facts. Firstly, the nutritional status of most of the patients was already poor at the time of admission. Secondly, albumin and pre-albumin are consistent markers of sepsis, so hypoalbuminemia was predominantly seen as patients developed septicaemia during their course of stay in ICU. And thirdly, patients with traumatic brain injury are frequently hyper-metabolic and hyper-catabolic; demonstrate many aspects of acute phase response [13-15], and often have depressed albumin concentration on admission and throughout much of their course of admission [15-20]. Hyponatremia following TBI can be due to syndrome of inappropriate ADH secretion (SIADH) or cerebral salt wasting syndrome [3,21], but it can also be due to decreased serum albumin following acute phase response [22]. Hypomagnesemia is commonly found in patients with hypoalbuminemia [23]. Hypomagnesemia is in turn commonly associated with hypokalaemia (refractory to potassium supplement) [24,25], hypocalcaemia [26], and hypophosphatemia [27]. While calcium has an integral role in excitation-contraction coupling of the airway smooth muscles, hypophosphatemia is associated with respiratory muscle weakness and difficulty in weaning from mechanical ventilation. Thus long-term deficiency of either of these electrolytes can increase the duration as well as the cost of ICU stay and also associated with patient morbidity as well as mortality. 

Lastly, creatinine is a measure of muscle mass and urea is the by-product of protein metabolism. In our study, both these variables were low in around 1/4th of the patients with traumatic brain injury. This can be explained by two understandings. First, patients whose nutritional status is already poor at the time of admission are less likely to have raised or normal urea and creatinine level. Second, metabolic acidosis is not a very uncommon finding in TBI [28] and chronic metabolic acidosis has been found to be associated with hypoalbuminemia and negative nitrogen balance [29]. While we found that decreased electrolyte level was also associated with negative nitrogen balance, but there was neither correlation between the two, neither any statistical significance. 

Limitations of the study

The sample size was small. Plasma and urine osmolarity could not be done that could differentiate between SIADH and cerebral salt wasting. Total calcium was used instead of corrected calcium or ionised calcium levels. As our CICU is six bedded, only the severe TBI patients were admitted and included in the study. So our study could very well miss the profile of mild to moderate TBI.

## Conclusions

Our study demonstrated that patients with TBI have a very strong propensity to develop electrolyte imbalance, particularly hyponatraemia and hypokalaemia. They are also prone to develop hypocalcaemia, hypomagnesemia and also hypophosphatemia and hypoalbuminemia. Hyponatremia may be the result of SIADH or cerebral salt wasting syndrome that is common in TBI. Hyponatremia in SAH results from the release of ANP (atrial natriuretic peptide) that can cause fluid and sodium loss in urine. Hypokalaemia may also be the result of urinary loss of potassium.

Hence it would be wise to choose appropriate fluid in TBI patients to prevent or treat any electrolyte imbalance at its earliest while keeping a wise watch over nitrogen balance and stop its fall down the ladder of protein catabolism as much as feasible.
